# Variables associated with general practitioners taking on serious mental disorder patients

**DOI:** 10.1186/1471-2296-10-41

**Published:** 2009-06-10

**Authors:** Marie-Josée Fleury, Jean-Marie Bamvita, Jacques Tremblay

**Affiliations:** 1 Department of Psychiatry, McGill University, Douglas Mental Health University Institute Research Centre (DMHUIRC), Quebec, Canada; 2 DMHUIRC, Montreal, Quebec, Canada

## Abstract

**Background:**

As part of community-based initiatives to strengthen integrated care and promote patient recovery, GPs are asked to play a greater part in treating serious mental disorder (SMD) patients. All current healthcare reforms favour the reinforcement of primary care. More information on enhancing the role of GPs in mental health would benefit policymakers, especially as regards SMD patients, where little research has been published as yet. This article assesses variables associated with GPs taking on SMD patients.

**Methods:**

The study, encompassing multiple sites, is based on a sample of 398 GPs, representative of the GP population in the Canadian province of Quebec. GPs were asked to answer a 143-item questionnaire on their socio-demographic and clinical practice profiles, patient characteristics, perceived inter-professional relationships and quality of care. Descriptive, bivariate and multivariate analyses were performed.

**Results:**

Our data highlighted that GPs currently followed up only a minority of SMD patients on a continuous basis and far fewer for both physical and mental health problems. A linear regression model that accounts for 43% of the variance was generated. The best variables associated positively with GPs taking on SMD patients were: frequency of referrals for joint follow-up with other resources, and involvement in post-hospitalization follow-up. Conversely, lack of expertise in mental health (related in our model to frequency of mental disorder patient transfer due to insufficient mental health training) is associated with a lower incidence of GPs taking on patients.

**Conclusion:**

As advocated in current healthcare reforms, our study confirms the need to promote greater GP involvement in integrated care models and enhance their training in mental health – thereby helping to reverse the trend among GPs of transferring SMD patients to specialized care. Patients with stable SMDs ought to have the same care access as the general population.

## Background

In light of efforts to improve healthcare efficiency, enhancing the integration of primary care within the mental healthcare system is strongly recommended [1-3]. It has been reported that in countries with more fully developed primary care, the healthcare system is more effective with regard to service accessibility, service continuity and patient outcomes [4-7]. The trend toward extending primary mental health care is related to the community-based movement in mental health whose objective is to promote patient recovery [8-10]. Over the past 40 years, deinstitutionalization has returned individuals with mental disorders to the community. Accordingly, general practitioners (GPs) are increasingly viewed as major partners in the mental healthcare system [1,11].

In recent years, many primary care models have proliferated, favouring collaboration, care continuity, and best-practices for the management of patients with chronic and complex problems (e.g., Wagner's Chronic Care Model; the patient-centered medical home approach) [12,13]. In Canada, two such examples in mental health are shared-care [14,15] and integrated service network models [15-17], which aim at improving care co-ordination among GPs, psychiatrists and multidisciplinary mental health providers, or within the healthcare system as a whole. These models usually include a broad spectrum of integration strategies and best practices such as clinical guidelines, electronic medical records, case management, capitation and performance incentives for GP remuneration, and patient self-management support [14,16,18]. In the province of Quebec, multidisciplinary group practices such as "family medicine groups" involving several GPs working closely together with nurses responsible for patient screening, follow-up, referral and patient registration are other innovations designed to improve service continuity and patient outcomes. It has been shown that these innovative integrated models not only improve care continuity but also more appropriately meet the needs of mental health patients living in the community [19,20].

Compared to specialized care, services provided primarily by GPs for patients with mental disorders are found to be more accessible, less stigmatizing and more comprehensive, since physical problems are managed along with mental disorders [21]. As the main entry point into the healthcare system [22,23], GPs play a pivotal role in screening, detecting and treating mental disorders [24,25]. In the course of a single year, about 80% of the population consults a GP, and between 20 and 40% of visits are related to mental health [26,27]. In Canada, of all patients with a mental disorder seeking help, 45% consult a GP while 25% consult other healthcare practitioners [22,25]. About 25% of patients with chronic psychosis see only their GPs [27]. Depression and anxiety are the predominant common problems in mental health patients seen by GPs [24,28,29]. In the context of current reforms designed to promote patient recovery and further deinstitutionalization, GPs are increasingly being asked to play a pivotal role with regard to stabilized serious mental disorder (SMD) patients (e.g., schizophrenia). This is a trend not only in Canada, but in most countries [2,30].

Although current reforms encourage GPs to manage more SMD patients, few studies have been published as yet on this subject [19,31,32]. Most studies involving GPs focus on common mental disorders (e.g. depression and anxiety), examine best practices for treatment and assess outcomes for various types of intervention and programs [12,14]. In the other hand, studies on SMD patients examine almost exclusively the mental health network, rarely including GPs' care [11]. To our knowledge, no prior study has investigated variables that promote or hinder the involvement of GPs with SMD patients, which is a prerequisite for the development of optimal integrated care models for these patients. Accordingly, this study is designed to test the association of GPs taking on SMD patients and multiple correlates such as GPs' socio-demographic profile, clinical practice, perceived inter-professional relationships and quality of care, and patient characteristics. Although based on the Quebec/Canada context, the findings from this study should be of wider relevance since primary mental health care in most of the industrialized countries share similar reform objectives (e.g., optimizing GPs' role, accessibly and continuity of care), and organizational and practice features (e.g., United Kingdom, Australia, Ireland) [33].

## Method

### Design and study population

This cross-sectional study was conducted among GPs practising in the province of Quebec, in Canada. Quebec has a population of 7.5 million, and 7,199 full-time GPs [34]. The study sites represented rural, semi-urban and urban territories (with or without a university-affiliated psychiatric hospital). In each of these sites, participants were selected in a variety of settings, including solo or group practices in private clinics, local community-based service centers (CLSCs), hospitals (acute, psychiatric or long-term), walk-in clinics, family medicine groups, and network clinics. CLSCs are local organizations that provide a broad range of health and psychosocial services, including mental health. Network clinics are similar to family medicine groups, except that patients are not registered with their GPs, and nurses act mainly as liaison agents. The sample list was provided by the Quebec Federation of General Practitioners (FMOQ), the professional union representing Quebec GPs. Recruitment took place from September 2006 to February 2007. Each participant was required to sign a consent form approved by the Douglas Mental Health University Institute research ethics board.

### Data collection process

As no prior questionnaire existed, a self-administered questionnaire including six main domains and 143 items, based on a literature review, was designed by the research team. It was validated by a multidisciplinary group of twenty experts (researchers, GPs and psychiatrists). The RAMQ (Régie de l'assurance maladie du Québec) 2006 data bank – the public register for all GPs' medical acts – was also used (e.g., number of GP medical acts, percentage of patients with mental disorders) for the purpose of comparison. The questionnaire was pre-tested with ten physicians not included in the study sample. Its structure reflects our goal to cover every possible aspect of GP practice in mental health, without exceeding a maximum of 30 minutes to complete the questionnaire. No financial incentive was offered to respondents.

The questionnaire covered six main domains: (1) GP socio-demographic and attitudinal profile, (2) patient characteristics, (3) clinical practice features, (4) collaboration between GPs and other medical or psychosocial mental health professionals, (5) GP perception of quality of mental health services, and (6) GP opinions about supportive strategies to be promoted for better care integration. It includes either categorical or continuous items or five-point Likert scales (1 = strongly disagree to 5 = strongly agree).

The questionnaire was sent by mail. Each questionnaire was assigned a tracking number and accompanied by support letters from the Quebec College of Physicians and FMOQ. There were three follow-ups. The first was conducted by mail. In the second, a nurse called the GPs. For the third, GPs were contacted by network medical administrators of the target territories. More information on the questionnaire and the sampling procedure can be found in another publication [35].

### Statistical analyses and definition of variables

Univariate, bivariate and multivariate analysis were performed on the questionnaire items linked to the dependent variable. The model was built using linear regression analysis. The proportion of SMD patients taken on by GPs, out of the total number of SMD patients seen, was the dependent variable, which was measured as a continuous variable. The term "taking on" patients goes further than seeing patients during a medical visit (one-time basis), and implies relational continuity and follow-up over time for the same or subsequent condition, including medical tests (physical and/or mental health), medication, side-effect monitoring, psychotherapy or any kind of psychosocial support. It was based on answers given by GPs to the following question: "Among patients seen with SMD in your medical practice weekly, what is the proportion of SMD patients you follow up on a continuous basis (i.e., accepted as your own patients)?" Mental disorders in the study were divided into two broad categories: (1) common mental disorders, which include anxiety, depression, adaptation disorders, personality disorders and substance abuse co-morbid disorders; and (2) SMD, which excludes the latter and for which three examples were provided: schizophrenia, bipolar disorder and delirious disorder.

Independent variables were organized in five sets related to the five first main dimensions of the questionnaire (identified above). Associations yielding a *p *value of less than 0.10 in bivariate analyses were considered for the multiple regression model. In each of the five variable sets, a partial model was constructed using the backward stepwise method (p ≤ 0.05). The final model was designed using the same technique of elimination by adjusting all the variables from the five sets. It was validated for goodness of fit, proportion of variance explained, and collinearity diagnostics.

## Results

### Sample

The sample comprised 398 GPs, for a response rate of 41%. More information is presented on the sampling procedure in another publication [35]. The sample was compared to non-respondent GPs for gender distribution, which yielded a non-significant result (χ^2 ^= 3.44, df = 1, P = 0.0637). Other important parameters were used to compare our sample with the overall GP population in Quebec [34,36,37]. Where data were available, tests were carried out comparing the GP population in Quebec and Canada [38]. As shown in Table 1, no significant difference was found.

**Table 1:** Comparison between our GP sample and the Quebec/Canada GP population

	Sample (%)	All Quebec GPs (%)	Sample versus all Quebec GPs			All Canadian GPs	Quebec GPs versus Canadian GPs		
			X^2^	df	p		X^2^	df	p
Age categories (years of age)			20.00	16	0.22		20.000	16	0.22
< 35	8.3	13.7				13.25			
35–44	32.9	27.5				30.95			
45–54	41.5	35.0				32.6			
55–64	14.6	18.3				17.35			
65+	2.8	5.5				5.85			
Gender distribution			3.44	1	0.06		1.32	1	0.250
Male	48.7	55.1				63.3			
Female	51.3	44.9				36.7			
Clinical setting									
Private medical offices	80.1	69.8	2.67	1	0.10				
CLSCs	23.6	27.3	0.24	1	0.63				
Hospitals	49.4	57.3	1.28	1	0.26				
Emergency services	17.3	25.2	1.93	1	0.16				
Practice area			1.34	1	0.25		2.49	1	0.11
Urban	66.3	74.9				84.2			
Rural	33.7	25.1				15.8			
Presence of a university hospital			1.29	1	0.26				
Yes	51.0	58.6							
No	49.0	41.4							
Income level from fee for service	65.0	74.0	1.90	1	0.17	51.0	1.28	1	0.257
Percentage of patients presenting with a mental disorder in the GP clientele	24.9	20.0	0.47	1	0.49				

### GP profiles

In Tables 2 and 3, pertinent information is provided on GP socio-demographic, clinical and inter-professional collaboration profiles regarding the management of SMD patients. The study showed that one quarter of GP consultations concerned mental health problems. Of all the patients visiting GPs for any mental health reasons, approximately one out of ten was found to consult for SMD. Only one third of SMD patients (34% ± 36) were followed up on a continuous basis by GPs. The long-term mental health management of these patients related mostly to medication follow-up (49% ± 39) and supportive therapy (35% ± 37), with GPs seeing them on average six times (± 5) a year. GPs estimated referring a majority of SMD patients (71% ± 3) mainly to emergency rooms and psychiatric services. They primarily referred those patients for advice on medication and diagnostic evaluation. When they instead transferred mental disorder patients (for any types of care and for either a short-term or a long-term period), the main reasons were case severity or complexity.

**Table 2:** General practitioner (GP) socio-demographic and clinical characteristics (n = 398)

GP average age [mean (SD)]	48 (± 9)
Hours spent on duty per week [mean (SD)]	43 (± 13)
Number of patients seen (or patient consultations) in a week for any reasons [mean (SD)]	90 (± 42)
Proportion of medical consultations related to mental disorders, both common and serious mental disorders [mean %]	25 (± 19)
Proportion of serious mental disorders (SMD) patients (e.g. schizophrenia, bipolar and delirious disorder) diagnosed among GP patient consultations related to mental disorders [mean % (SD)]	12 (± 13)
Proportion of patients with common mental disorders (CMD, i.e. depression and/or anxiety disorders, adaptation disorders, personality disorders, substance abuse) among GP patient consultations related to mental disorders [mean % (SD)]	88 (± 42)
Proportion of SMD patients taken on by GPs (i.e., accepted as the GP own patients, GPs assuming continuous follow-up over time) among total GP consultations of SMD patients (i.e. one-time basis and GPs' own patients) [mean % (SD)]	34 (± 36)
Proportion of GP consultations of SMD patients taken on related to the following reasons [mean % (SD)]:	
medication follow-up	49 (± 39)
supportive therapy	35 (± 37)
psychotherapy	8 (± 21)
Number of times GPs received their SMD patients (i.e. patients taken on) annually [mean (SD)]	6 (± 5)
Number of patients visiting GPs per week for mental disorders, both common and serious disorders [mean (SD)]	23 (± 19)

**Table 3:** General practitioner (GP) inter-professional collaboration features (n = 398)

Number of patients referred per week to other resources for any types of care (among patients visiting GPs per week for mental disorders, both common and serious disorders) [mean (SD)]	5 (± 5)
Proportion of serious mental disorders (SMD) patients referred to other resources (among all SMD patients visiting GPs weekly, either those on a one-time basis or the GPs own patients) (%) [mean % (SD)]:	71 (± 34)
Among all GPs referring SMD patients, proportion of GPs who referred SMD patients for the following reasons [mean %]:	
advice on medication	84
diagnostic evaluation	82
Among patients visiting GPs with SMD, proportion referred by GPs (for any types of care) to the following resources [mean % (SD)]:	
Emergency room	27 (± 36)
Psychiatric services	22 (± 32)
Psychologists in private practice	5 (± 16)
CLSC:	
Mental health team	11 (± 24)
Psychosocial services	8 (± 20)
Voluntary sector (e.g. day centers and mutual self-help group)	4 (± 15)
Crisis center	4 (± 13)
Among GPs reporting transferring mental disorder patients (i.e., all GP mental disorder consultations, both SMD and common mental disorders), proportion of GPs who transferred patients occasionally and often (for any types of care, and for either a short-term or a long-term period) to other resources for the following reasons [mean %]:	
severity of the disorder	93.6
case complexity	92.1
lack of support from psychiatrists	62.7
insufficient mental health training	58.9
lack of interest in mental health	17.5
insufficient financial incentives	18.0

### Variables associated with GPs taking on SMD patients

The final multiple linear regression model is shown in Table 4, consisting of six variables organized in three sets. The most significantly associated variable was negatively related to taking on SMD patients: frequency of transfer of patients with mental disorders, owing to GPs' insufficient mental health training. The six variables included in the model accounted for 43% of the variance in the dependent variable. The model fit was significant (F = 45.88; p < 0.001).

**Table 4:** Variables independently associated with GPs taking on SMD patients

		Beta	*t *test	p value
Inter-professional relationship profile	Frequency of GP referrals for joint follow-up (of any types) with other resources for mental disorder patient care	5.49	3.76	0.002
	Frequency of GP involvement in post-hospital follow-up (related to both either patients or psychiatric professionals initiative) for mental disorder patient care	2.83	2.15	0.029
Mental health clinical practice profile	Proportion of SMD patients visiting GPs (total consultations, including one-time basis – walk-in clinics, or follow-up by GPs) on all patient consultations	0.61	5.45	< 0.001
	Proportion of medical visits related to SMD patient medication follow-up (on all SMD patient consultations)	0.26	5.87	< 0.001
	Proportion of medical visits related to SMD patient supportive therapy (on all SMD patient consultations)	0.19	4.24	< 0.001
Lack of expertise in mental health	Proportion of GPs who transfer mental disorder patients (out of all GP mental disorder consultations, and for any types of care and for either a short-term or long-term period) owing to insufficient mental health training	-10.60	-7.06	< 0.001

F = 45.88 (P < 0.001); R^2 ^= 0.43

*DEPENDENT VARIABLE*: number of serious mental disorder (SMD) patients taken on (i.e., accepted as the general practitioners' (GPs) own patients, GPs assuming continuous follow-up over time) by GPs among all patients seen with SMD (total GP consultations with SMD patients).

## Discussion

As found in other studies [28,32,37,39], our research findings showed that only a minority of SMD patients are managed primarily by GPs. Three sets of variables were associated with GPs taking on SMD patients: (1) their level of expertise toward treating those patients; (2) their inter-professional relationship feature; and (3) their clinical practice profile. Those three sets of variables, especially the first one that contains the most strongly associated variable in the model, should be considered as significant hindering or enabling factors for optimizing primary mental healthcare service planning.

Consistent with our findings, various studies have cast doubt on the ability of GPs to detect and treat more complex forms of mental disorders, particularly major depression with suicidal tendencies, schizophrenia, and bipolar disorders [28,40,41]. They have also highlighted GP discomfort with such patients. GPs either consider these disorders too specialized for routine primary care, deeming their skills and experience inadequate for effective diagnosis and treatment, or they position themselves as complementary to specialized care, treating essentially physical problems [19,23]. All of these conditions encourage GPs to transfer SMD patients to specialized care (i.e., psychiatric departments of acute-care or psychiatric hospitals, or emergency rooms).

Nevertheless, when GPs practice in integrated primary care models such as in shared-care or a patient-centered medical home approach, they are reportedly more at ease with managing patients with mental disorders [12,14,42]. This is confirmed by our findings: joint follow-up with other resources and involvement in post-hospital follow-up were found to be the second and third most important variables associated with GPs taking on these patients. Research showed that when SMD patients are successfully managed and stabilized by psychiatrists, GPs are more comfortable following them in the community with medication and/or supportive therapy, jointly with mental health teams, as required [43-46]. SMD patients generally need continuous community follow-up of varying intensity over time. They usually have numerous bio-psycho-social needs that require teamwork to avoid relapse and help adapt to a recovery-oriented life [47,48].

Along with the need for psychiatric team assistance, GPs who offer medication and supportive therapy follow-up and have a greater volume of SMD patients were also found, in our study, to be more likely to take on SMD patients. This is consistent with previous research [12,23,32] highlighting links between mental health knowledge and training, and the ability of GPs to manage these patients. Conversely, lack of both knowledge and training are major factors resulting in the transfer of patients by GPs to specialized care. A higher volume of SMD patient consultations would be expected to result in greater GP willingness to take them on. This further leads us to hypothesize that some GPs may specialize in the follow-up of these patients, being more able to manage both patient medication follow-up and supportive therapy. When all conditions favour SMD patient management by GPs, our findings also show that GPs apparently offer good continuity of care, seeing these patients on average six times a year. Half the time, SMD patients were followed-up by GPs either solely for their physical problems or for both physical and mental health problems

There are many reasons explaining why only a minority of GPs take on SMD patients. Such patients are deemed to require more care and time, more frequent visits, and be more difficult to treat [46,49]. Often, they have concurrent diagnoses (e.g., substance abuse) and interrelated physical or social problems [50-52]. As for GPs, their poor collaboration with psychiatry services, their busy schedules and the competing demands of other patients are other impending factors [7,14,53]. The historical separation between psychiatry and primary care [11], and GPs' limited training or experience with effective team practice [21] may also explain their reluctance to take on these patients – especially if they consider hospital psychiatric teams to be more appropriate. But none of this would suggest GPs' removal from the treatment equation of those patients. SMD patients are in great need of adequate physical care and mental health follow-up as they face higher risks of interrelated morbidity. Moreover, as psychiatric teams are usually concentrated more in urban settings, GPs are often the sole available source of care. This is the case in Quebec where almost half of the psychiatrists practice in the Montreal metropolitan area, and where in more remote regions, specialized care is scarce [54].

## Conclusion

While our model yields various strong and interesting associations, the study has certain limitations. First, it has a cross-sectional design, which does not permit causal inference as in experimental or longitudinal studies. Second, as the data collected are from GP self-reporting, the results must be viewed as an approximation of actual GP practice. Third, our questionnaire was complex, which may have discouraged some GPs from participating. As a result, the response rate was limited, but was not substantially lower than that reported in other surveys involving GPs [36,46,55]. Finally, no data was collected on GPs' adequacy in treating mental-disorder patients, which is considered a major issue giving rise to conflicting results in the literature [12]

In the context of current reforms designed to enhance healthcare efficiency and support SMD patients' integration in the community, our findings sustain the development of integrated care models favouring service co-ordination, exchange of expertise between bio-psycho-social professionals and healthcare lines of services, and reinforcement of GPs' mental-health training. Our data show that GPs currently follow up only a minority of SMD patients on a continuous basis, and far fewer for both their physical and mental health problems. However, GPs may play a pivotal role in taking on patients with stable SMDs, if they have psychiatrists' and mental health team support. Lack of expertise in mental healthcare was also found to be a strong impediment. Without psychiatrists' and mental health team support or sufficient expertise in mental health, GPs are likely to transfer SMD patients to specialized care.

In the Canadian context of high GP shortages, increasing the development of mental-health integrated care models could nevertheless be a major challenge. Patients with stable SMDs ought to have the same care access as the general population, however, and services that are the least stigmatizing. Group practice models such as family medicine groups with nurses working closely with GPs to assist in patient screening and follow-up could serve as the basis for more refined integrated care models in Quebec's mental healthcare system. Improvements in access to direct communications with psychiatrists or shared-care initiatives, for diagnostic and therapeutic consultations, and to mental health teams, for patient case management of various intensities, should also be considered. For managing chronic and complex illnesses such as SMDs, various studies [18,56] have indicated the major positive impact of comprehensive and continued multimodal strategies (e.g. clinical guidelines, electronic medical records, financing incentives, medical education sessions), which should be further encouraged.

## Competing interests

The authors declare that they have no competing interests.

## Authors' contributions

MJF designed and monitored the study. JMB performed the statistical analysis, in collaboration with JT. Both MJF and JMB wrote a draft of the article. JT assisted in the study design and commented the article. All authors read and approved the final manuscript.

## Pre-publication history

The pre-publication history for this paper can be accessed here:


